# Effects of inclusion of cetirizine hydrochloride in β-cyclodextrin

**DOI:** 10.1007/s10847-018-0808-y

**Published:** 2018-05-04

**Authors:** Magdalena Paczkowska, Mikołaj Mizera, Kornelia Lewandowska, Maciej Kozak, Andrzej Miklaszewski, Judyta Cielecka-Piontek

**Affiliations:** 1 Department of Pharmaceutical Chemistry, Faculty of Pharmacy, Poznan University of Medical Sciences, Grunwaldzka 6, 60-780 Poznan, Poland; 2 Department of Molecular Crystals, Institute of Molecular Physics, Polish Academy of Sciences, Smoluchowskiego 17, 60-179 Poznan, Poland; 3 Department of Macromolecular Physics, Adam Mickiewicz University, Umultowska 85, 61-614 Poznan, Poland; 4 Institute of Materials Science and Engineering, Poznan University of Technology, Jana Pawła II 24, 60-965 Poznan, Poland

**Keywords:** Cetirizine hydrochloride, β-Cyclodextrin, Quantum chemical calculations, Chemical stability, Solubility, Permeability

## Abstract

Following the preparation of inclusion complex of cetirizine (CTZ) and β-cyclodextrin (β-CD), the compound was investigated to assess the possibility of modifying the physicochemical properties (solubility, release, stability, permeability) of CTZ after complexation that are vital for subsequent formulation studies involving the said complex. Changes in FT-IR/Raman spectra, DSC thermograms and XRD diffractograms confirmed the formation of a CTZ–β-CD system. Hydrophilic interaction chromatography with a DAD detector was employed to determine alterations of the CTZ concentration during studies following complexation. An analysis of a phase-solubility diagram of c_CTZ_ = *f*c_β-CD_ indicated a linear rise in the solubility of CTZ as the concentration of β-CD increased. The inclusion of CTZ in a system with β-CD significantly reduced the instability of CTZ in the presence of oxidizing factors. It was also found that regardless of the pH of the acceptor fluids used in the release studies an increase was observed in the concentration of CTZ in CD system compared to its free form. The ability to permeate artificial biological membranes manifested by CTZ after complexation was enhanced as well. In summary, CD has significant potential to mask the bitter taste of CTZ and to counter the instability induced by oxidizing factors.

## Introduction

Cetirizine hydrochloride (CTZ) is a second-generation H_1_-receptor antagonist antihistamine drug [1]. It is used to treat the symptoms of allergies, chronic urticaria, hay fever, angioedema, hives and as a treatment adjunct in asthma [2]. CTZ belongs to the second generation of antihistamines that are less able to cross the blood–brain barrier and therefore do not cause side effects such as sedation, drowsiness, and decreased cognitive processing [3–6]. Cetirizine is a racemic mixture of two enantiomers: levocetirizine and dextrocetirizine. Although levocetirizine shows 30-fold greater efficiency against the H_1_-receptor, the majority of preparations contain a racemic mixture of two enantiomers because no correlation has been found between the content of the optical isomers and the perception of a bitter taste. Efforts to find ways of masking the bitterness of cetirizine have been dictated by two important issues: the possibility of using the drug in pediatric patients depending on its therapeutic safety and effectiveness in the context of a growing tendency for allergic reactions in that subpopulation, and the need to select taste-masking substances able to reduce the susceptibility of CTZ to degradation under the influence of oxidizing factors. One of the impurities listed in Pharmacopoeial guidelines is 1,4-bis-[(4-chlorophenyl)phenylmethyl]piperazine, formed as a result of the action of an oxidizing factor. The formation of related impurities as a consequence of CTZ oxidation or subsequent degradation of a specified Pharmacopoeial impurity has also been reported [7–9].

Considering that it is necessary to mask the taste of CTZ and to ensure its stability, cyclodextrins (CDs) may be recommended as auxiliary substances with the potential to satisfy those requirements. They are cyclic oligomers composed of six, seven or eight glucose monomers, linked by α-1,4-glucose bonds, referred to as α-, β- or γ-cyclodextrins, respectively. It has been reported in some studies that CDs can form inclusion (host–guest) complexes with several pharmaceutical substances containing hydrophobic molecules [10–12]. When used as complexing agents, CDs can also help to increase drug solubility and to enhance drug permeability through the membrane barrier thus improving the bioavailability of the guest molecule [13–19]. Moreover, there have been reports of employing CDs as carrier systems for targeted drug delivery [20–23].

**Fig. 1:**
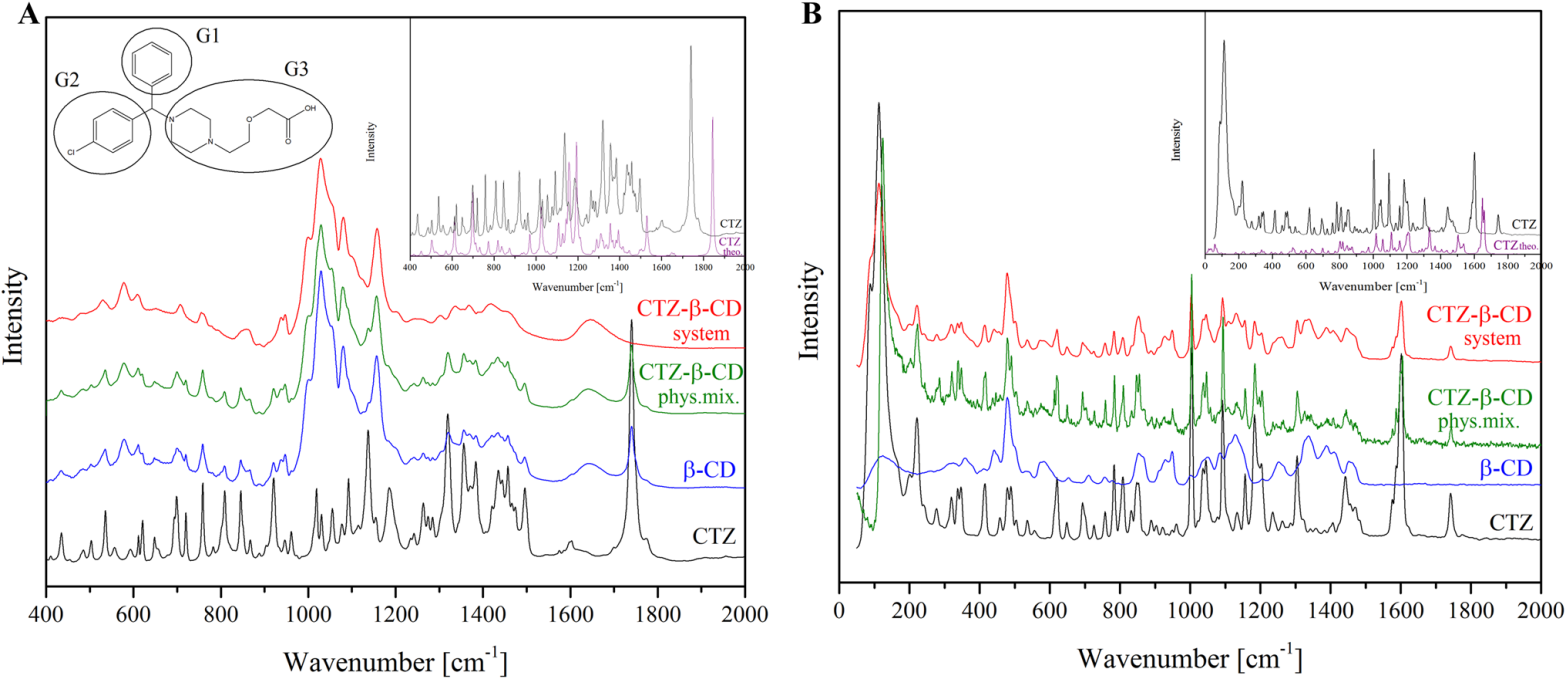
FT-IR (**a**) and Raman spectra (**b**) of CTZ, β-CD, CTZ–β-CD physical mixture and CTZ–β-CD system, and calculated IR CTZ spectra (B3LYP/6-31G) (CTZ theo.) with chemical structure of cetirizine showing main groups (*G1* phenyl group, *G2* chlorophenyl group, *G3* piperazinyl ethoxy acetic acid)

With regard to masking the unpleasant taste of CTZ, successful attempts reported in the literature involved complexation with β-CDs [24–26]. Several patent descriptions refer to pharmaceutical formulations of CTZ containing CDs as agents for the reduction of its bitter taste [27, 28]. Fanara et al. presented the formation of solutions of CTZ and CD that diminished the unpalatable flavor as a result of the pre-formation of a drug-cyclodextrin complex. Friesen et al. incorporated CTZ into multiparticulates (55 wt% glyceryl mono-, di- and tri-behenates, COMPRITOL 888, and 10 wt% poloxamer, PLURONIC F-127) to provide a dissolution-retarded form of the drug and then chewable tablets containing a dissolution-retarded form of the CTZ and β-cyclodextrin complex. It should be emphasized that all of the reports on the preparation of the CTZ–β-CD complex referred to co-precipitation as a preparation method. The solvents were water [25, 26, 29, 30], a mixture of water and methanol [31], and DMSO [24]. The preparation methods presented in the literature necessitate, due to the prospect of using the complex in solid drug forms, the removal of solvents and monitoring their volatile residues. Considering the predicted possibility of incorporating CTZ into the structure of CD, three domains have been specified: the phenyl and chlorophenyl groups as well as the piperazinyl ethoxy acetic moiety (Fig. 1a). The first two are hydrophobic and may contribute to inclusion complexation. The third is hydrophilic and as such it is favored by the exterior hydrophilic group of CD. The structure of a cyclodextrin complex was determined by Stojanov et al. during studies using NMR spectroscopy [25]. They confirmed the stereochemistry of CTZ complexes with α-, β- and γ-CD, and indicated two possible CD binding sites in the CTZ structure: the phenyl and chlorophenyl rings. However, so far no reports have described the effect of complexing CTZ and β-CD on the properties (solubility, stability, release, permeability) important for future formulation work on such complexes. Therefore, the aim of the present work was to prepare and characterize a cetirizine inclusion complex with β-cyclodextrin regarding changes in the solubility, stability, release and permeability of cetirizine resulting from the formation of the said complex. The methods used for identification studies of the CTZ–β-CD complex included Fourier transform infrared (FT-IR) and Raman spectroscopies, X-ray powder diffraction (XRPD) as well as differential scanning calorimetry (DSC). Changes in the concentration of CTZ during the evaluation of its solubility, stability, release and permeability were determined with a HILIC method [7].

## Materials and methods

### Materials

Cetirizine hydrochloride (purity > 98%) in bulk substance was supplied by India PVT LTD (Navi Mumbai, India), while β-cyclodextrin (purity > 98%) by Sigma-Aldrich (Poznan, Poland). Hydrochloric acid, sodium hydroxide solvent, hydrogen peroxide, potassium dihydrogen phosphate, potassium bromide and all other chemicals were obtained from Avantor Performance Materials (Gliwice, Poland). Acetonitrile of an LC grade was supplied by Merck KGaA (Darmstadt, Germany) and formic acid (100%) by Avantor Performance Materials (Gliwice, Poland). High-quality pure water was prepared using an Elix SA 67120 Millipore purification system (Warsaw, Poland).

### Preparation of the CTZ–β-CD system

CTZ was kneaded with a mixture of β-CD and water at a molar ratio of 1:1. After drying at 313 K, the substances were ground to powder. The CTZ–β-CD system was kept in an atmosphere of controlled humidity.

### Characterization of the CTZ–β-CD system

During identification studies of the CTZ–β-CD system, spectroscopic (FT-IR, Raman, XRPD) and thermal (DSC) techniques were used. A HILIC method was applied for the measurement of CTZ concentration changes during studies of solubility, chemical stability, release and permeability through biological membranes.

#### Fourier transform infrared spectroscopy (FT-IR)

Infrared spectra for CTZ, β-CD, the CTZ–β-CD physical mixture and the CTZ–β-CD system were recorded with a Fourier transform infrared FT-IR Bruker Equinox 55 spectrometer equipped with a Bruker Hyperion 1000 microscope. All samples were measured in an absorption mode, in a frequency range of 400–2000 cm^−1^ and at a resolution of 4 cm^−1^. To analyze changes in positions and intensity in the experimental spectra of the CTZ–β-CD system, quantum chemical calculations based on DFT with B3LYP functional and 6-31G(d,p) basis set were conducted to obtain theoretical spectra of CTZ. The analysis of changes in experimental spectra and determination of binding domains of API in system was conducted according to theoretical spectrum of CTZ. All calculations were performed by using the Gaussian 09 package [32]. The scale factor applied to theoretical spectra was 0.964.

#### Raman spectroscopy

Raman scattering spectra for CTZ, β-CD, CTZ–β-CD physical mixture and the CTZ–β-CD system were obtained with a LabRAM HR800 spectrometer (HORIBA Jobin Yvon) with the laser (He–Ne) excitation λ_exc_ = 633 nm. All of the spectra were an average of 500 scans with a resolution of 4 cm^−1^. In each case the power of the laser beam at the sample was less than 1 mW to avoid damage to the sample.

#### X-ray powder diffraction (XRPD)

For base sample analysis and the confirmation of system formation, an X-ray powder diffraction (XRPD) technique was used. The obtained patterns were collected by a PANalitycal Empyrean system with a Cu lamp (1.54056 Å). The measurements were carried out in the scanning range between 3° and 50° at 2*θ* range using a step size of 0.017° and a step time of 15 s/step with source parameters of 45 kV and 40 mA.

#### Differential scanning calorimetry (DSC)

DSC analyses of CTZ, β-CD, CTZ–β-CD physical mixture and the CTZ–β-CD system were performed by using a DSC 204 Phoenix differential scanning calorimeter (Netzsch, Germany). The samples of 2 mg were enclosed in aluminum cells, and heated at a scanning rate of 5 K min^−1^ from 273 to 523 K in a helium atmosphere with a flow rate of 40 mL min^−1^. The obtained DSC data were analyzed (determination of enthalpy, phase transition temperatures, etc.) using the computer program TA (Netzsch).

#### HILIC method

The Ultimate 3000 LC system (Dionex Thermoline Fisher Scientific, Germany) included a photodiode array detector was used which was equipped with a high-pressure pump (UltiMate 3000), an autosampler (UltiMate 3000) and a DAD detector (UltiMate 3000). Data were collected and processed by Chromeleon software version 7.0 from Dionex Thermoline Fisher Scientific (US). Chromatographic separation was performed on a Poroshell 120 Hilic (4.6 × 150 mm, 2.7 µm) column using a mobile phase composed of acetonitrile-0.1% formic acid (20:80 *V*/*V*) at a flow rate of 1.0 mL min^−1^. The injection volume was 5.0 µL. The wavelength of detection was controlled at 235 nm. The HILIC method was validated in regards to CTZ determination, including selectivity of determination in the presence of degradation products forming during oxidation [7].

### Studies of the CTZ–β-CD system

#### Phase solubility studies of CTZ–β-CD system

The phase-solubility profile of the CTZ–β-CD system was determined according to the method of Higuchi and Connors [33]. Excess amounts of CTZ were added to samples of β-CD (0.5–10.0 mmol L^−1^), followed by water, and kneaded for 1 h at 298 K, within which time the equilibrium of the CTZ system was achieved. Samples containing a physical mixture of CTZ–β-CD were ground for 1 h at 298 K. The concentrations of CTZ in each sample were determined by an HILIC method. Phase-solubility studies were performed in triplicate; from which the apparent 1:1 stability constant was calculated. The phase-solubility profile of CTZ was obtained by plotting the solubility of CTZ as a function of β-CD concentration. The following equation was applied to establish the apparent stability constant for CTZ–β-CD:

1 $${K_{1:1}}=\frac{{{\text{Slope}}}}{{{{\text{S}}_0}(1 - {\text{slope}})}}$$

where S_0_ is the intercept and slope is the angular coefficient of the fitted straight line.

#### Stability studies of the CTZ–β-CD system

Due to the degradation of CTZ in the presence of an affecting factor (H_2_O_2_), the impact of the oxidizing factor concentration on CTZ in free form and after complexation was investigated. Samples of CTZ–β-CD for stability studies were prepared by dissolving an accurately weighed 39.2 mg (containing 10.0 mg of free CTZ) in 20.0 mL of an equilibrated solution to 353 K in glass-stoppered flasks, where samples were exposed to oxidizing factor in different concentrations. At specified times, samples of the reaction solutions (1.0 mL) were collected, instantly cooled, and chromatographic determination was conducted. The concentration changes of CTZ and CTZ–β-CD were studied in aqueous solutions in hydrogen peroxide, with the concentration of H_2_O_2_ ranging from 2 to 5% [7]. To verify k_obs_ determined for the degradation of CTZ in free and complexed forms, a parallelism test was used. The following equations were applied to establish the significant differences of stability plots of CTZ in free and complexed forms:

2 $${t_0}=\frac{{{a_1} - {a_2}}}{{{S_{{a_1} - {a_2}}}}}$$

3 $${S_{{a_1} - {a_2}}}=\sqrt {\frac{{\mathop \sum \nolimits_{{i=1}}^{{{n_1}}} {{\left( {{y_{i1}} - {{\bar {y}}_{i1}}} \right)}^2}+\mathop \sum \nolimits_{{i=1}}^{{{n_2}}} {{\left( {{y_{i2}}+{{\bar {y}}_{i2}}} \right)}^2}}}{{{n_1}+{n_2} - 4}} \times \left( {\frac{1}{{\mathop \sum \nolimits_{{i=1}}^{{{n_1}}} {{\left( {{x_{i1}} - {{\bar {x}}_1}} \right)}^2}}}+\frac{1}{{\mathop \sum \nolimits_{{i=1}}^{{{n_2}}} {{\left( {{x_{i2}} - {{\bar {x}}_2}} \right)}^2}}}} \right)}$$

where $\mathop \sum \nolimits_{{i=1}}^{{{n_1}}} {\left( {{y_{i1}} - {{\bar {y}}_{i1}}} \right)^2}$ and $\mathop \sum \nolimits_{{i=1}}^{{{n_2}}} {\left( {{y_{i2}}+{{\bar {y}}_{i2}}} \right)^2}$ are the sum of the squares of the difference in the deviation of the regression curve determined experimentally.

#### In vitro release study of the CTZ–β-CD system

The release of CTZ from the CTZ–β-CD system was studied with the help of an Agilent 708-DS dissolution apparatus. A standard paddle method was used at 310 ± 0.5 K with a stirring speed of 50 rpm. CTZ in free form, a physical mixture of CTZ–β-CD and the CTZ–β-CD system weighed into gelatin capsules were placed in spring to prevent flotation of the capsule on the surface of the liquid. The so-obtained samples were placed in 500 mL media of simulated gastric fluids (pH 1.2) and phosphate buffer (pH 5.0, 6.2 and 7.4) simulating the gastro-intestinal environment. Release samples (5.0 mL) were collected at specified time intervals with the replacement of an equal volume of temperature-equilibrated media and filtered through a 0.45 µm membrane filter. The concentrations of CTZ in acceptor solutions were determined by using a HILIC method [7]. The reported value s are arithmetic means of six measurements.

The model proposed by Moore and Flanner to compare the release profiles is based on two-factor values, *f*_1_ and *f*_2_ [34, 35]. The difference factor (*f*_1_) measures the percent error between two curves over all time points and *f*_2_ is a logarithmic transformation of the sum-squared error of differences between the test *T*_*j*_ and reference *R*_*j*_ system over all time points according to the formulas below:

4 $${f_1}=\frac{{\mathop \sum \nolimits_{{j=1}}^{n} \left| {{R_j} - {T_j}} \right|}}{{\mathop \sum \nolimits_{{j=1}}^{n} {R_j}}} \times 100$$

5 $${f_2}=50~ \times \log \left( {{{\left( {1+\left( {\frac{1}{n}} \right)\mathop \sum \limits_{{j=1}}^{n} {{\left| {{R_j} - {T_j}} \right|}^2}} \right)}^{ - \frac{1}{2}}} \times 100} \right)$$

where *n* is the sampling number, *R*_*j*_ and *T*_*j*_ are the percents dissolved of the reference (CTZ) and test products (CTZ–β-CD system) at each time point *j*. Dissolution profiles are similar when the *f*_1_ value is close to 0 and *f*_2_ is close to 100 (FDA guidelines suggest that two profiles are similar if *f*_2_ is between 50 and 100).

#### Permeability studies

Differences in the gastrointestinal permeability of CTZ in free and complexed forms were investigated by using a PAMPA method (parallel artificial membrane permeability assay). The system consisted of a 96-well microfilter plate and a 96-well filter plate and was divided into two chambers: a donor at the bottom and an acceptor at the top, separated by a 120-µm-thick microfilter disc coated with a 20% (w/v) dodecane solution of a lecithin mixture (Pion, Inc.). Solutions of CTZ and the CTZ–β-CD system (0.30 mmol L^−1^) in water were prepared in a different 96-well filter plate and added to the donor compartments. The donor solution was adjusted to pH 5.0, 6.2 or 7.4 (NaOH-treated universal buffer). The plates were put together and incubated at 310 K for 4 h in a humidity-saturated atmosphere. The concentrations of CTZ in free and complexed forms were determined by using the HILIC method in the donor and acceptor compartments after 4 h of incubation.

The apparent permeability coefficient (P_app_) was calculated by using the Eq. 6 as follows:

6 $${P_{app}}=\frac{{ - \ln \left( {1 - \frac{{{C_A}}}{{{C_{equilibrium}}}}} \right)}}{{S \times \left( {\frac{1}{{{V_D}}}+\frac{1}{{{V_A}}}} \right) \times t}}$$

where *V*_*D*_ is the donor volume, *V*_*A*_ the acceptor volume, *C*_*equilibrium*_ the equilibrium concentration ${C_{equilibrium}}={{{C_D} \times {V_D}+~{C_A} \times {V_A}} \mathord{\left/ {\vphantom {{{C_D} \times {V_D}+~{C_A} \times {V_A}} {{V_D}+{V_A}}}} \right. \kern-0pt} {{V_D}+{V_A}}}$, *C*_*D*_ the donor concentration, *C*_*A*_ the acceptor concentration, *S* the membrane area and *t* is the incubation time (in seconds). Compounds with P_app_ < 1 × 10^−6^ cm s^−1^ are classified as ones with low permeability and those with P_app_ > 1 × 10^−6^ cm s^−1^ as ones with high permeability [36]. To verify that P_app_ determined for permeability of CTZ in free and complexed forms was statistically different, ANOVA test was used.

## Results and discussion

The results presented in this paper were discussed with the focus on two areas: the procedure of identifying the CTZ–β-CD system and the evaluation of the effect of complexation on the solubility, stability, release and permeability of CTZ through an artificial membrane simulating gastrointestinal permeation as compared to the free form of CTZ.

The system was obtained via a co-grinding technique and its formation was a spontaneous and repeatable process. Previous identification studies of the CTZ–β-CD system relied on NMR spectroscopy [25]. The present work aimed to confirm the formation of the system by using spectroscopic (FT-IR/Raman, XRPD) and thermal (DSC) methods, thus adopting a novel approach to the identification of the CTZ–β-CD system.

The types of bands, their location and intensity were determined for a CTZ molecule in free form and compared with theoretical spectra based on quantum-chemical calculations performed with the use of the B3LYP functional and 6-31G(d,p) as a basis set. The most important characteristic vibration bands of CTZ were defined (Table 1). In the CTZ–β-CD spectra, a decrease in the intensity of bands corresponding to vibrations between C–C–C in the phenyl and chlorophenyl rings and changes in the vibrations of C–H in those rings were observed. Interestingly, an analysis of changes in the bands of CTZ in complexed form indicated the disappearance of bands at 1185/1183 cm^−1^ in the FT-IR/Raman spectra corresponding to the bending vibrations between C–O–H as well as in C–O in piperazinyl ethoxy acetic acid, and a decrease in the intensity of a broad band at 1384 cm^−1^ in the FT-IR spectrum corresponding to the bending vibrations between C–O in piperazinyl ethoxy acetic acid (Fig. 1a, b). These results confirmed the participation of two different (hydrophilic and hydrophobic) domains in a CTZ molecule in the interaction with β-CD. For physical mixture of the CTZ–β-CD abovementioned changes were not observed.

**Table 1:** Selected characteristic vibronic features of CTZ

Theory scaled (cm^−1^)	IR (cm^−1^)	Raman (cm^−1^)	Approximate description
589	612		C–O–H *b* + C–O–C *b*
**617**	**648**	**649**	**C**–**C**–**C** ***b*** **in G1 and G2**
672	697	694	O–H *w* + *br* G2 ring
**687**		**726**	**C**–**H** ***w*** **in G1 and G2** + ***br*** **G1 and G2**
**745**	**758**	**757**	**C**–**H** ***w*** **in G1 and G2** + ***br*** **G1 and G2**
773	783	784	*br* G3 + C–H *r* at G3 + C–H *w* at G1 and G2
**789**	**808**	**808**	***br*** **G1 and G2** + **C**–**H** ***w*** **in G1**
**813**	**846**	**848**	**C**–**C** ***s*** **at G3** + **C**–**O** ***s*** **at G3** + **C**–**N** ***s***
829		851	C–H *w* in G1
844	867		C–H *r*
**936**	**961**	**961**	**C**–**O** ***s*** **at G3**
979		1004	C–C–C *b* in G1 and G3
988	1018		C–C *s* in G1 and G2 + C–N *s*
1019		1045	*br* G1 + C–C *s* at G3
1069	1093	1093	C–H *r* in G1 and G2 + C–Cl *s*
1102	1137		C–N *s* + C–H *w* + C–O *s*
**1114**		**1157**	**C**–**N** ***s*** + **C**–**C** ***s*** **at G3** + **C**–**H** ***r***
**1151**	**1185**	**1183**	**C**–**O**–**H** ***b*** + **C**–**O** ***s*** **at G3**
1168		1204	C–H *r*
1227		1235	C–H *w*
1243	1242		C–H *w*
**1266**	**1263**		**C**–**H** ***t***
**1285**		**1304**	**C**–**H** ***t***
**1306**	**1320**		**C**–**H** ***w*** **at G3**
**1344**	**1384**		**C**–**O**–**H** ***b*** **at G3** + **C**–**C** ***s*** **at G3** + **C**–**H** ***w*** **at G3**
1420		1407	C–H *sc* at G3
1449		1443	C–H *sc* at G3
**1474**	**1495**		**C**–**H** ***sc***
1599	1605	1603	C–C *s* in G1 and G2
1777	1739	1743	C=O *s*
2799			C–H *s* between G1 and G2
2843			C–H *s* at G3
2906	2869		C–H *s* at G3
2986	2951		C–H *s* sym. and asym.
3087	3054		C–H *s* sym. and asym. in G1 and G2
3611	3439		O–H *s*

The most important characteristic vibrational bands of CTZ involved in the interaction with B-CD are marked as bold

*s* Stretching, *b* bending, *r* rocking, *w* wagging, *sc* scissoring, *t* twisting, *oop* out of the plane, *br* breathing, *G1* phenyl group, *G2* chlorophenyl group, *G3* piperazinyl ethoxy acetic group

**Fig. 2:**
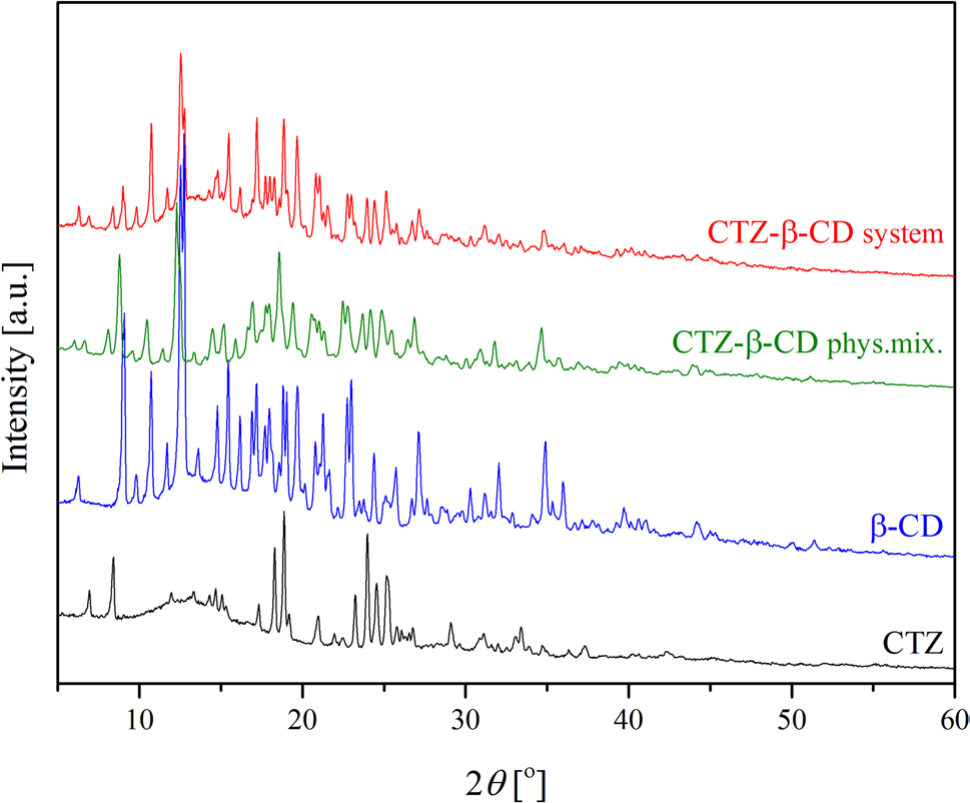
XRPD powder diffraction patterns of CTZ, β-CD, CTZ–β-CD physical mixture and CTZ–β-CD system

The X-ray powder diffraction patterns for the individual components, physical mixture and the system are shown in Fig. 2. For all analyzed data the presence of crystalline form was verified. The diffraction data confirmed the formation of the system. This is interpreted in Fig. 2 as the sum relation of the spectral lines of both initial components CTZ and β-CD, the presence of which is confirmed by their peak positions. Since X-ray powder diffraction corroborated the results obtained from FT-IR and Raman spectroscopy, it may also be considered a method for the verification of incorporation (inclusion).

**Fig. 3:**
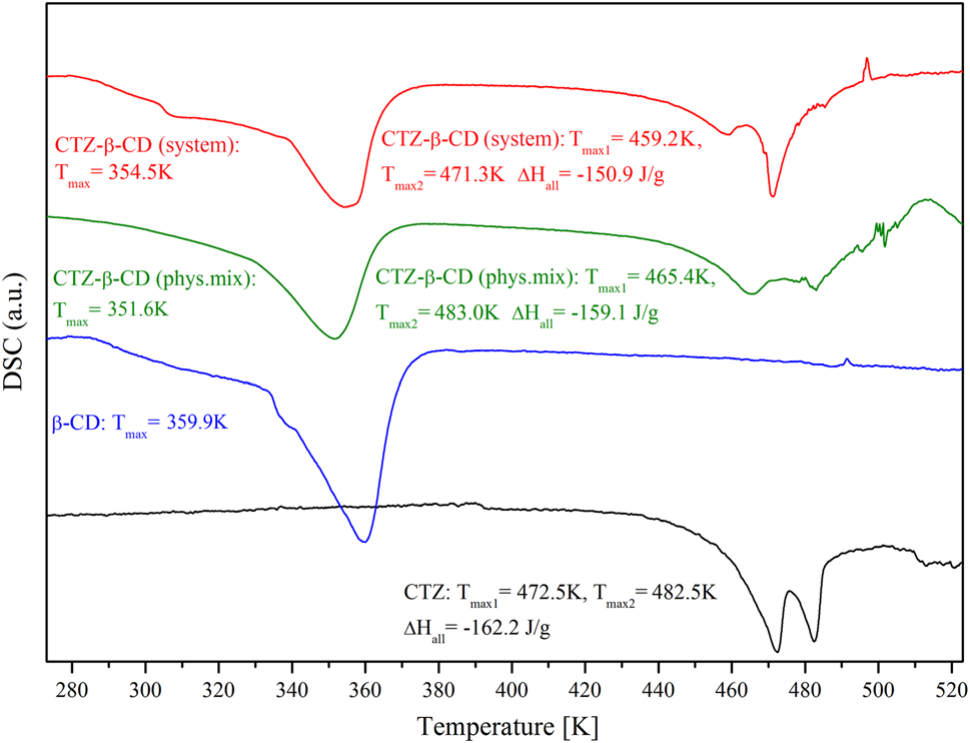
DSC thermograms of CTZ, β-CD, CTZ–β-CD physical mixture and CTZ–β-CD system

The DSC thermograms collected for CTZ, β-CD, CTZ–β-CD physical mixture and the CTZ–β-CD system are presented in Fig. 3. In the DSC thermogram obtained for a pure sample of CTZ, two distinct endothermic peaks are visible. The first peak was observed with a minimum at T_1_ = 472.6 K, while the second at T_2_ = 482.6 K. The thermal profile of β-CD exhibited a broad endothermic peak with a minimum at 360.0 K, which corresponded to dehydration resulting from the presence of water molecules trapped inside the β-CD cavity [37]. In the thermal profile of the CTZ–β-CD system, the shape of the endothermic peak at 354.6 K changed and the two peaks (at T_1_ = 472.6 K and at T_2_ = 482.6 K), corresponding to CTZ, also showed changed shapes.

Previous studies reported the importance of CTZ–β-CD system formation for obtaining a masking agent for the bitter taste of CTZ without referring to other effects of system formation. Hence, the pioneering aspect of the present work consists in investigating the physicochemical properties of CTZ after complexation.

The HILIC method was used for investigations of phase-solubility, chemical stability, release and permeability of CTZ in free and system forms. The changes of CTZ concentrations in the presence of its main product formed during oxidization were evaluated using HILIC method. The HILIC method was validated for this purpose (Table 2).

**Table 2:** Validation parameters of CTZ determination by HILIC method

Parameter	Results			
	pH 1.2	pH 5.0	pH 6.2	pH 7.4
Selectivity				
Peak symmetry factor (in range of 0.8–1.5 required)	1.030	1.066	1.025	1.027
Absence of interfering substances	Confirmed	Confirmed	Confirmed	Confirmed
Limit of detection (LOD): LOD = 3 SD/*a* (mg mL^−1^)	0.0097	0.6471	0.6413	1.2500
Limit of quantification (LOQ): LOQ = 10 SD/*a* (mg mL^−1^)	0.0294	1.9609	1.9436	3.7879
Linearity: y = *a*x + *b*				
*a* ± S_a_	8.24 ± 0.11	4.84 ± 0.81	6.02 ± 0.11	5.34 ± 0.12
*b* ± S_b_	Insignificant	6.49 ± 0.26	6.08 ± 0.13	5.04 ± 0.12
Correlation coefficient (*r*)	0.9998	0.9985	0.9982	0.9971
Range of linearity (mg mL^−1^)	0.01–3.59	0.64–5.16	0.64–4.38	0.57–5.72
Accuracy				
Recovery (95–105% required) (%)	96.07	95.31	95.39	95.31
Precision				
Concentration (mg mL^−1^)	0.5900	5.1600	4.2800	5.7200
Average of six injections (mg mL^−1^)	0.5668	4.9182	4.0829	5.4517
SD	0.0004	0.0070	0.0014	0.0113
RSD (< 5% required)	0.0781	0.1429	0.0335	0.2177

Where SD is the mean of standard deviations of determinations in the lower range of linearity and *a* is the directional coefficient of the plotted linear function

S_*a*_ standard deviation of the slope, S_*b*_ standard deviation of the intercept, *t* calculated values of Student’s *t* test, t_α,f_ = 2.228 critical values of Student’s *t* test for degrees of freedom f = 10 and significance level α = 0.05

**Fig. 4:**
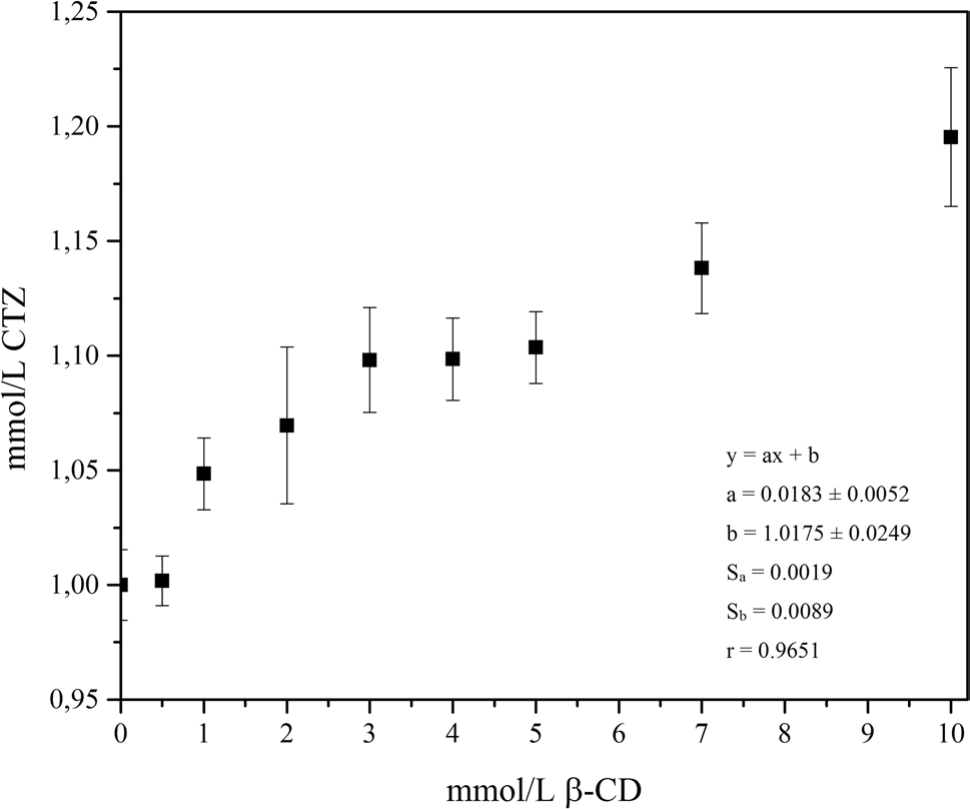
Phase-solubility diagram of c_CTZ_ = *f*c_β-CD_ with statistical evaluation

The first of the properties associated with CD is its suitability for use as a solubilizer. The phase-solubility study was conducted in the mixture c_CTZ_ = *f*c_β-CD_ for a range of β-CD concentration of 0–10 mmol L^−1^. The profile of the phase-solubility of CTZ is presented in Fig. 4. The solubility of CTZ increased linearly as a function of CD concentration, which corresponded with A-type phase-solubility profile according to Higuchi and Connors [33]. Parameters of the regression were calculated for f = n − 2 degrees of freedom with a = 0.05. The calibration curve for was described by the equation y = ax + b. The apparent stability constant was 0.0183 mmol^−1^. It may be suggested that a 20% increase in CTZ solubility (from 1.0 to 1.2 mmol L^−1^) resulted from its incorporation into β-CD as well as from the adhesion of solubilizing CD to CTZ molecules in the area of the phenyl and chlorophenyl rings [25].

According to the BCS classification (*biopharmaceutics classification system based on differences of solubility and permeability*), CTZ belongs to Class III, whose members are characterized by high solubility but low permeability. Admittedly, as a result of applying cetirizine in salt form it was possible to include it in that category of drugs. It should be stressed that the solubility of CTZ is further enhanced after incorporation of its hydrophobic domains into the cavity in a CD molecule.

Another area of studies on the effects of CTZ–β-CD system formation focused on the evaluation of β-CD impact on the chemical stability of CTZ. Previous studies showed the significant lability of CTZ in the presence of an oxidizing factor [7–9]. In order to compare the stability of CTZ in free form and after complexation with β-CD, the stability studies were conducted in the presence of an oxidizing factor (H_2_O_2_, c = 2–5%, T = 353 K). The introduction of CTZ into the cavity of β-CD did not influence the mechanism of CTZ degradation (Table 3). The degradation of CTZ in free as well as in complexed form was a pseudo-first-order reaction described by the Eq. 7:

**Table 3:** Kinetic parameters of CTZ degradation in free form and in complex with β-CD

Temp. (K)	H_2_O_2_ (%)	CTZ	CTZ–β-CD system	t_0_
		Kinetic parameters		
353	2	k ± Δk = (7.51± 0.68) × 10^−7^ (s^−1^) S_k_ = 1.01 × 10^−4^ r = 0.9965 n = 7	k ± Δk = (5.77± 0.61) × 10^−7^ (s^−1^) S_k_ = 8.98 × 10^−5^ r = 0.9954 n = 7	4.64
353	3	k ± Δk = (1.13± 0.12) × 10^−6^ (s^−1^) S_k_ = 2.90 × 10^−4^ r = 0.9876 n = 7	k ± Δk = (7.32± 0.11) × 10^−7^ (s^−1^) S_k_ = 1.57 × 10^−4^ r = 0.9913 n = 7	4.39
353	4	k ± Δk = (1.36± 0.15) × 10^−6^ (s^−1^) S_k_ = 2.18 × 10^−4^ r = 0.9951 n = 7	k ± Δk = (9.86± 0.18) × 10^−7^ (s^−1^) S_k_ = 2.62 × 10^−4^ r = 0.9866 n = 7	4.02
353	5	k ± Δk = (1.75± 0.24) × 10^−6^ (s^−1^) S_k_ = 3.56 × 10^−4^ r = 0.9921 n = 7	k ± Δk = (1.02± 0.33) × 10^−6^ (s^−1^) S_k_ = 4.88 × 10^−4^ r = 0.9582 n = 7	4.36

*t*_0_ parameter of parallelism test

7 $$\ln \left( {{C_{CTZ}}} \right)=\ln {\left( {{C_{CTZ}}} \right)_0} - {k_{obs}}$$

The semi-logarithmic plots were linear and their slopes were equal to the rate constants of the reactions with the negative sign (− k_obs_). To verify that k_obs_ determined for both CTZ degradations were insignificant the parallelism test was used. β-CD had a stabilizing effect on the chemical stability of CTZ. In the HPLC chromatograms, peaks originating from an oxidized degradation product did not occur.

**Fig. 5:**
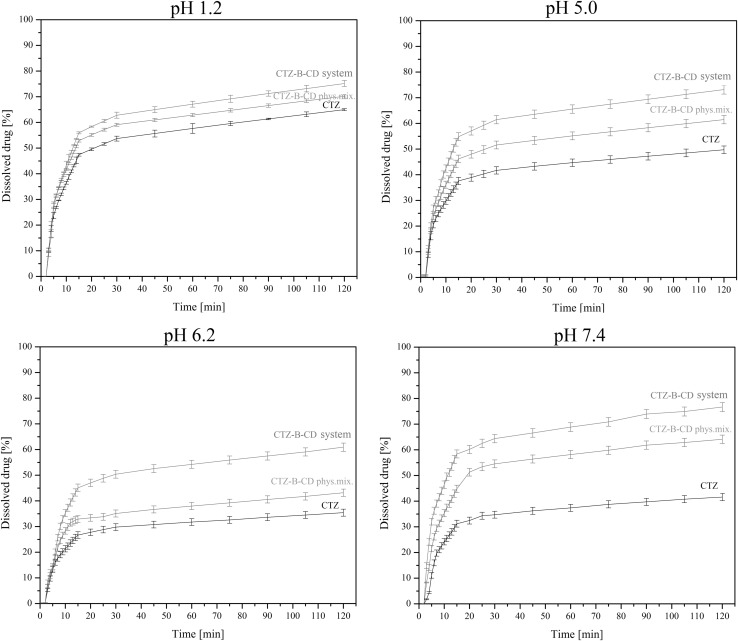
Release profiles at various pH for CTZ, CTZ–β-CD physical mixture and CTZ–β-CD system

There have been numerous reports of the use of CD as a release modifier for selected molecules [14–16]. The release profiles for CTZ and the CTZ–β-CD system are presented in Fig. 5. The release profiles of CTZ and the CTZ–β-CD system were compared in simulated gastric fluids (pH 1.2) and phosphate buffer (pH 5.0, 6.2 and 7.4). An analysis of the release profiles showed that CD significantly enhanced CTZ solubility. This was observed on obtaining a physical mixture and occurred without changing the shape of the release curves. The release rate of the system was enhanced for all tested pH values when compared to CTZ in free form. The release process of the system started faster and quickly reached a peak concentration. For all tested values of pH, the difference factor *f*_1_ exceeded 15 and the *f*_2_ factor was below 50, which reflected major differences between the CTZ and CTZ–β-CD system release profiles and the strong influence of β-CD on the rate of CTZ release.

The last aspect of interest in the influence of CD on CTZ after complexation was changes in the permeability of CTZ through an artificial membrane after complexation determined against a reference standard that was the permeability of CTZ in free form. The greatest permeability of CTZ was at pH 1.2 [(1.59 ± 0.17) × 10^−5^ cm s^−1^], followed by pH 5.0 [(1.55 ± 0.22) × 10^−5^ cm s^−1^] and pH 7.4 [(1.33 ± 0.17) × 10^−5^ cm s^−1^]. The lowest permeability occurred at pH 6.2 [(1.30 ± 0.17) × 10^−5^ cm s^−1^]. For the CTZ–β-CD system, the highest permeability was at pH 1.2 [(1.69 ± 0.13) × 10^−5^ cm s^−1^], followed by pH 7.4 [(1.67 ± 0.16) × 10^−5^ cm s^−1^], pH 5.0 [(1.60 ± 0.13) × 10^−5^ cm s^−1^] and, the lowest, at pH 6.2 [(1.50 ± 0.16) × 10^−5^ cm s^−1^]. The significance of differences between results obtained for those forms CTZ and CTZ–β-CD were evaluated by using ANOVA test (pH 1.2–1.59 × 10^−5^ vs 1.69 × 10^−5^ cm s^−1^, p = 0.0001 < α = 0.05, pH 5.0–1.55 × 10^−5^ vs 1.60 × 10^−5^ cm s^−1^, p = 0.0036 < α = 0.05; pH 6.2–1.30 × 10^−5^ vs 1.50 × 10^−5^ cm s^−1^, p = 0.00016 < α = 0.05; pH 7.4–1.33 × 10^−5^ vs 1.67 × 10^−5^ cm s^−1^, p = 0.00002 < α = 0.05). The hydrophilicity of cetirizine salt pointed to diffusion as the main mechanism of permeation. Regardless of the pH of donor solutions, CTZ after complexation displayed greater permeability compared to its free form, which was confirmed statistically. The fact that the results of the permeability tests did not correlate with the findings of the release experiments suggested that the modification of release and the improvement of permeability were separate phenomena relative to the effect of incorporating phenyl and chlorophenyl rings into a β-CD cavity.

## Conclusion

Within the scope of the present study, the authors have demonstrated the possibility of modifying the physicochemical properties of CTZ (studied by using FT-IR, Raman, XRPD and DSC methods) as a result of its interaction with CD in the course of dry kneading. The observable effects of the process were the increased release of CTZ irrespective of pH, the enhanced chemical stability of CTZ under oxidizing conditions and the greater permeability of CTZ through biological membranes. No correlation was found between pH and either CTZ release from CTZ-βCD system or CTZ permeability. This finding should be given careful consideration in future work on the preparation of pharmaceutical formulations using so-obtained CTZ–β-CD system.
